# 86. Antibiotic-resistant gram-negative bacterial infections among persons with or without a prior positive test for SARS-CoV-2 in 10 U.S. sites, 2020

**DOI:** 10.1093/ofid/ofac492.011

**Published:** 2022-12-15

**Authors:** Sandra N Bulens, Julian E Grass, Nadezhda Duffy, Jigsa Tola, Jesse T Jacob, Gillian Smith, Elisabeth Vaeth, Ghinwa Dumyati, Hsioa Che Looi, Erin C Phipps, Kristina Flores, Christopher Wilson, Daniel Muleta, Christopher A Czaja, Jennifer Driscoll, Ruth Lynfield, Sean M O'Malley, Meghan Maloney, Nicole Stabach, Joelle Nadle, Rebecca Pierce, Heather Hertzel, Alice Guh

**Affiliations:** CDC, Atlanta, GA; CDC, Atlanta, GA; CDC, Atlanta, GA; CDC, Atlanta, GA; Emory University School of Medicine, Atlanta, GA; Georgia Emerging Infections Program, Atlanta, GA, Atlanta, Georgia; Georgia Emerging Infections Program, Atlanta, GA; Foundation for Atlanta Veterans Education and Research, Decatur, GA; Atlanta Veterans Affairs Medical Center, Decatur, GA, Atlanta, Georgia; Maryland Department of Health, Baltimore, Maryland, Baltimore, Maryland; University of Rochester Medical Center, Rochester, New York; New York Rochester Emerging Infections Program at the University of Rochester Medical Center, Rochester, New York, Rochester, New York; University of New Mexico, Albuquerque, NM; New Mexico Emerging Infections Program, Santa Fe, NM, Albuquerque, New Mexico; University of New Mexico, Albuquerque, NM; New Mexico Emerging Infections Program, Santa Fe, NM, Albuquerque, New Mexico; Tennessee Department of Health, Nashville, Tennessee; Tennessee Department of Health, Nashville TN, Antioch, Tennessee; Colorado Department of Public Health and Environment, Denver, CO, Denver, Colorado; Colorado Department of Public Health and Environment, Denver, CO, Denver, Colorado; Minnesota Department of Health, St. Paul, Minnesota; Minnesota Department of Health, St. Paul, MN, St. Paul, Minnesota; Connecticut Department of Public Health, Hartford, Connecticut; Connecticut Department of Public Health, Hartford, CT, Hartford, Connecticut; California Emerging Infections Program, Oakland, California; Oregon Health Authority; Portland, OR., Portland, Oregon; Oregon Public Health Division, Oregon Health Authority; Portland, OR., Portland, Oregon; CDC, Atlanta, GA

## Abstract

**Background:**

The Centers for Disease Control and Prevention’s Emerging Infections Program (EIP) conducts active laboratory- and population-based surveillance for carbapenem-resistant Enterobacterales (CRE), extended spectrum beta-lactamase-producing Enterobacterales (ESBL-E), and carbapenem-resistant *Acinetobacter baumannii* (CRAB) in 10 U.S. sites. To describe the impact of the COVID-19 pandemic on the epidemiology of these antibiotic-resistant gram-negative bacteria (AR-GNB), we assessed characteristics of AR-GNB patients with and without a prior SARS-CoV-2 positive (SC2+) viral test.

**Methods:**

In 2020 among EIP catchment-area residents, an incident CRAB or CRE case was defined as the first isolation of *A. baumannii* complex, *Escherichia coli, Enterobacter cloacae* complex*, Klebsiella aerogenes, K. oxytoca, K. pneumonia*, or *K. variicola* in a 30-day period resistant to ≥1 carbapenem (excluding ertapenem for CRAB) from a normally sterile site or urine. An incident ESBL-E case was defined as the first isolation of *E. coli, K. pneumonia*, or *K. oxytoca* in a 30-day period resistant to any third-generation cephalosporin and non-resistant to all carbapenems from a normally sterile site or urine. Patient charts were reviewed.

**Results:**

Of 3904 AR-GNB cases with data available, 163 (4%) had a prior SC2+ test (85 ESBL-E, 70 CRE, and 8 CRAB). Median time from the most recent SC2+ test to AR-GNB culture date was 20 days (IQR 1–48 days). AR-GNB cases with a SC2+ test versus those without were more likely to be Black, non-Hispanic than another race/ethnicity (31% vs 15%; P< 0.0001), aged ≥65 years (62% vs 52%; P=0.0139), and to have prior healthcare exposures (63% vs 49%; P=0.0003) and indwelling devices (51% vs 28%; P< 0.0001). They were also more likely to have bacteremia (24% vs 11%; P< 0.0001), pneumonia (6% vs 1%; P< 0.0001) and be hospitalized around the time of their AR-GNB culture (67% vs 36%; P< 0.0001); median time from SC2+ test to hospital admission was 0.5 day (IQR 0–29.5 days).

**Conclusion:**

AR-GNB infections preceded by a SC2+ test were rare but more severe and associated with more healthcare risk factors. This underscores the need for continued infection prevention and control practices and monitoring of these infections during the COVID-19 pandemic.

**Disclosures:**

**Ghinwa Dumyati, MD**, Pfizer: Grant/Research Support.

